# Current and emerging therapies in *IDH*-mutant glioma

**DOI:** 10.1016/j.neurot.2026.e00913

**Published:** 2026-05-04

**Authors:** Vihang Nakhate, Gilbert Youssef, Patrick Y. Wen

**Affiliations:** a Center for Neuro-Oncology, Dana-Farber Cancer Institute, Boston, MA, USA; b Harvard Medical School, Boston, MA, USA; c Center for Tumors of the Nervous System, Mass General Brigham Cancer Institute, Boston, MA, USA

**Keywords:** *IDH*-mutant glioma, Low grade glioma, Vorasidenib, IDH inhibitor

## Abstract

Isocitrate dehydrogenase (*IDH*)-mutant gliomas constitute a distinct molecular subtype of diffuse gliomas, characterized by unique biology and relatively favorable clinical outcomes. However, despite their more indolent initial course, these tumors ultimately develop treatment resistance and remain incurable. Standard treatment approaches have relied on surgery followed by radiation and alkylating chemotherapy, which provide meaningful disease control but are associated with cumulative neurocognitive toxicities. The oral mutant IDH inhibitor vorasidenib recently became the first targeted therapy available for *IDH*-mutant glioma. Based on results of the randomized phase 3 INDIGO trial, vorasidenib was approved by the US Food and Drug Administration in 2024 as a first-line treatment option for grade 2 *IDH*-mutant glioma following surgery. In this review, we summarize the current therapeutic paradigm for *IDH*-mutant glioma, including the use of radiation and chemotherapy as well as the evolving role of mutant IDH-targeted therapy. We also highlight emerging therapeutic strategies, including approaches targeting key biologically informed vulnerabilities such as DNA damage repair pathways, cell-cycle and metabolic dependencies, tumor-associated hypermethylation, and anti-tumor immune activation. Collectively, these advances reflect a rapidly evolving treatment landscape driven by improved understanding of *IDH* biology, and hold promise to overcome therapeutic resistance and improve patient outcomes.

## Introduction

Diffuse gliomas are the most common primary malignant brain tumors in adults [1]. The identification in 2008 of isocitrate dehydrogenase (*IDH)* mutations among a subset of gliomas led to a paradigm shift in glioma classification, prognostication, and management. Adult diffuse gliomas are now classified into three molecular subtypes according to the 2021 World Health Organization Classification of Tumors of the Central Nervous System (WHO CNS5): glioblastoma (GBM), *IDH*-wild type (grade 4); astrocytoma, *IDH*-mutant (grade 2–4); and oligodendroglioma, *IDH*-mutant and 1p/19q-codeleted (grade 2–3) [2]. *IDH*-mutant gliomas comprise a biologically and clinically distinct entity characterized by relatively favorable prognosis compared to *IDH*-wild type GBM. Although their clinical behavior ranges from indolent to aggressive depending on histologic grade and molecular features, *IDH*-mutant gliomas are uniformly infiltrative and ultimately treatment-resistant. Nearly two decades of scientific and clinical investigation into *IDH*-mutant gliomas has thus far culminated in the US Food and Drug Administration's (FDA) 2024 approval of mutant IDH (mIDH) inhibitor vorasidenib, paving the way to a new era of targeted therapy in glioma. In this review article, we outline the current therapeutic paradigm for *IDH*-mutant glioma, including the evolving roles of chemoradiation and mIDH inhibitors. We also examine emerging treatment strategies under investigation and consider potential avenues for future therapeutic advances.

## *IDH*-mutant glioma biology and classification

**Fig. 1:**
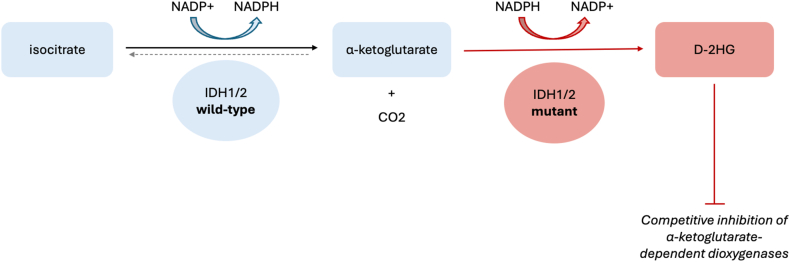
**Schematic representation of wild-type and mutant IDH1/2 enzyme function.** Wild-type IDH1/2 catalyze the reversible decarboxylation of isocitrate to α-ketoglutarate with reduction of NADP^+^ to NADPH. Mutant IDH1/2 acquire neomorphic activity that reduces α-ketoglutarate to D-2-hydroxyglutarate (D-2HG) while oxidizing NADPH to NADP^+^. Accumulated D-2HG competitively inhibits α-ketoglutarate–dependent dioxygenases and drives oncogenesis. Abbreviations *D-2HG* D-2-hydroxyglutarate, IDH isocitrate dehydrogenase, *NADP*^+^, nicotinamide adenine dinucleotide phosphate (oxidized form), *NADPH* nicotinamide adenine dinucleotide phosphate (reduced form), *CO*_*2*_ carbon dioxide.

The wild-type human IDH enzyme exists in three isoforms. IDH1 and IDH2 function independently as homodimers to catalyze the reversible oxidation of isocitrate to α-ketoglutarate while reducing NADP + to NADPH (Fig. 1). IDH3 catalyzes the same reaction irreversibly in the citric acid cycle while reducing NAD + to NADH [3]. Mutations in the active sites of *IDH1* and *IDH2* were initially identified in approximately 12% of histologically classified GBMs and in approximately 70–80% of histologically classified grade 2–3 diffuse astrocytomas and oligodendrogliomas [4,5]. They have also been identified in acute myeloid leukemia (AML), cholangiocarcinoma, and chondrosarcoma [6]. More than 90% of *IDH*-mutant gliomas harbor the somatic heterozygous *IDH1* R132H arginine-to-histidine point mutation, while the remainder harbor other point mutations at *IDH1* R132 or *IDH2* R172 loci [[7], [8], [9]].

mIDH enzyme gains neomorphic activity, converting NADPH and α-ketoglutarate into NADP+ and the pathologic oncometabolite D-2-hydroxyglutarate (D-2HG) (Fig. 1). D-2HG accumulates in *IDH*-mutant cells and is thought to contribute to oncogenesis by competitive inhibition of α-ketoglutarate-dependent dioxygenases, resulting in epigenetic dysregulation, altered cellular differentiation, impaired DNA repair, and suppression of anti-tumor immunity [[10], [11], [12], [13]]. In particular, D-2HG inhibits ten-eleven translocation (TET) family demethylases resulting in histone and DNA hypermethylation, particularly on CpG islands, and the glioma CpG island methylator phenotype (gCIMP) that promotes gliomagenesis [14,15].

**Fig. 2:**
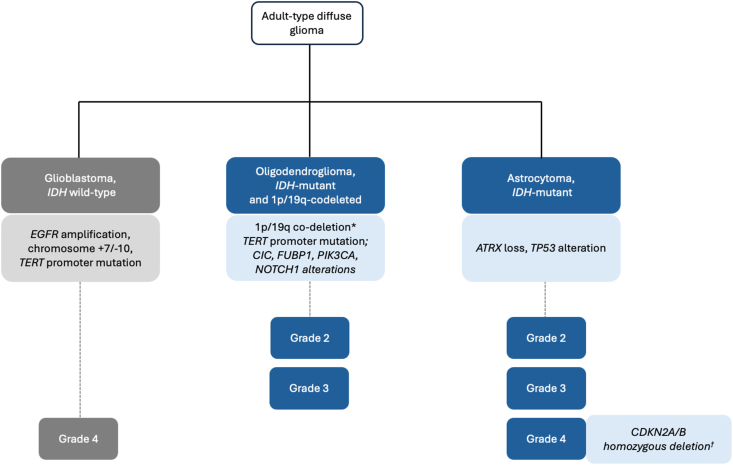
**Molecular changes associated with adult-type diffuse gliomas.** According to the 2021 World Health Organization Classification of Tumors of the Central Nervous System, adult-type diffuse gliomas are classified based on histologic and molecular features into: glioblastoma, *IDH* wild-type; astrocytoma, *IDH*-mutant; and oligodendroglioma, *IDH*-mutant and 1p/19q-codeleted. 1p/19q-codeletion is required for diagnosis of oligodendroglioma, *IDH*-mutant and 1p/19q-codeleted. Other commonly associated molecular alterations are shown. Tumor grade is designated based on both histologic and molecular features. Histologic features associated with tumor grades are not shown. ∗required for diagnosis. †CNS WHO grade 4 can be designated based on detection of *CDKN2A/B* homozygous deletion or based on the presence on histology of microvascular proliferation or necrosis.

Additional molecular changes distinguish *IDH*-mutant astrocytoma from *IDH*-mutant oligodendroglioma (Fig. 2). *IDH*-mutant oligodendrogliomas are defined by an unbalanced translocation resulting in co-deletion of the 1p and 19q chromosomal arms, and typically achieve telomere maintenance by *TERT* promoter activating mutations. Most *IDH*-mutant astrocytomas harbor *TP53* alterations, and most achieve telomere maintenance through loss of *ATRX* function [16,17]. Tumor grades of 2–4 (astrocytoma) or 2 to 3 (oligodendroglioma) are designated based on histologic and molecular features including anaplasia and increased mitotic activity (grade 3), presence of microvascular proliferation or necrosis (grade 4 in astrocytoma; grade 3 in oligodendroglioma), or homozygous loss of *CDKN2A/B* (grade 4 in astrocytoma) [18].

Clinically, *IDH*-mutant gliomas occur in younger patients (typically second to fifth decades of life) than *IDH*-wild type GBM, and are more epileptogenic [1,19]. On MRI they may be contrast enhancing, non-enhancing, or both. The presence of enhancement in recurrent *IDH*-mutant glioma tumors generally signifies a more aggressive phenotype and poorer prognosis [18]. Oligodendrogliomas are more indolent than astrocytomas and carry a more favorable prognosis [20].

## Current treatment approaches

### Surgery, radiation, and chemotherapy

The mainstay of initial management in *IDH*-mutant glioma is maximal safe surgical resection. Supramaximal resection of non-enhancing tumor when safe may confer survival benefit particularly in grade 2 *IDH*-mutant astrocytoma [21]. Surgery is often followed by radiation and systemic alkylating chemotherapy, typically temozolomide or PCV (procarbazine, lomustine and vincristine) depending on grade and histology. Vorasidenib (discussed in the next section) is used in a select subset of patients after surgery to delay the need for chemoradiation.

Much of the prospective randomized trial evidence for chemoradiation in *IDH*-mutant gliomas predates the molecular era. In grade 2 glioma (astrocytoma or oligodendroglioma) with age ≥40 years or sub-total resection/biopsy, adjuvant radiation followed by PCV demonstrated progression-free survival (PFS) and overall survival (OS) benefit compared to radiation alone in the randomized NRG Oncology/RTOG 9802 trial [22]. Post-hoc genomic analysis in a subset of patients found that only *IDH*-mutant gliomas derived this survival benefit, whereas *IDH* wild-type tumors did not (Table 1) [23]. It bears mention that the aforementioned patient population (age ≥40 years or sub-total resection/biopsy) was historically considered “high risk” for early progression in histologically classified tumors, but these parameters may not adequately characterize risk in molecularly defined tumors.

**Table 1:** Median PFS and OS for *IDH*-mutant gliomas in select randomized trials. Outcomes for indicated molecular subgroups are shown.

Tumor	Population	Treatment	Median OS	Median PFS	Trials
Oligodendroglioma, *IDH*-mutant, 1p/19q-codeleted, grade 2	Newly diagnosed	RT + PCV (vs RT)	NR (13.9y)	NR (5.8y)	RTOG 9802
Oligodendroglioma, 1p/19q-codeleted, grade 3	Newly diagnosed	RT + PCV (vs RT)	13.2y, 14.2y (7.3y, 9.3y)	9.8y, 13.1y (2.9y, 4.2y)	RTOG 9402, EORTC 26951
Astrocytoma, *IDH*-mutant, grade 2	Newly diagnosed	RT + PCV (vs RT)	11.4y (4.3y)	10.4y (3.3y)	RTOG 9802
Astrocytoma, *IDH*-mutant, grade 3	Newly diagnosed	RT + TMZ^a^ (vs RT)	12.5y (6.0y)	7.0y (3.4y)	CATNON (EORTC 26053-22054)
*IDH*-mutant glioma grade 2	Untreated non-enhancing residual or recurrent, after surgery	Vorasidenib (vs placebo)	NA	27.7 m (11.1 m)	INDIGO
*IDH*-mutant astrocytoma grade 3	Recurrent	Eflornithine + CCNU (vs CCNU)	34.9 m (vs. 23.5 m)	15.8 m (vs. 7.2 m)	STELLAR^b^

Abbreviations *EORTC* European Organization for Research and Treatment of Cancer, *m* months, *NA* not available, *NR* not reached, *OS* overall survival, *PCV* procarbazine lomustine vincristine, *PFS* progression-free survival, *RT* radiation, *RTOG* Radiation Therapy Oncology Group, *TMZ* temozolomide, *vs* versus, *y* years.

a Median PFS and OS figures reflect *IDH*-mutant tumors that received radiation and adjuvant temozolomide vs. radiation without adjuvant temozolomide.

b In the intention-to-treat analysis of the STELLAR trial among histologic anaplastic astrocytoma, there was no OS difference between the two treatment arms. In subgroup analysis of *IDH*-mutant astrocytoma grade 3, there was a clinically meaningful OS benefit in the combination arm.

In grade 3 oligodendroglioma, the RTOG 9402 and EORTC 26951 randomized trials demonstrated PFS and OS benefit among patients randomized to radiation and PCV chemotherapy compared to radiation alone, with particular benefit in 1p/19q-codeleted tumors (Table 1) (1p/19q-codeletion has since been subsumed into the definition of *IDH*-mutant oligodendroglioma) [[24], [25], [26]]. Temozolomide (TMZ) has a more favorable toxicity profile than PCV and is considered a reasonable alternative in *IDH*-mutant oligodendroglioma, though no published randomized studies have compared the two regimens. In a retrospective analysis of anaplastic oligodendroglioma, among 1p/19q-codeleted tumors PCV alone (without radiation) was associated with improved disease control but not improved OS compared to TMZ alone [27]. In a separate retrospective cohort of 1p/19q-codeleted grade 3 oligodendroglioma treated with radiotherapy, PCV was associated with improved unadjusted short-term OS compared to TMZ; however this difference was no longer statistically significant upon adjustment for age and extent of resection [28]. In a recent retrospective study of *IDH*-mutant 1p/19q-codeleted grade 3 oligodendroglioma, radiation with PCV was associated with improved OS compared to radiation with TMZ including after adjusting for known confounders [29]. The ongoing prospective randomized CODEL study comparing radiotherapy plus PCV with radiotherapy plus TMZ is expected to more definitively address this question (NCT00887146).

For grade 3 *IDH*-mutant astrocytoma, the randomized CATNON trial demonstrated survival benefit of radiation followed by twelve cycles of adjuvant TMZ compared to radiation alone, with no additional benefit from concurrent TMZ during radiation [30,31]. Finally, grade 4 *IDH*-mutant astrocytomas reflect a newly defined entity in the WHO 2021 classification with limited prospective treatment data, though retrospective cohorts have been characterized [32]. Standard of care treatment often mirrors that of glioblastoma or grade 3 astrocytoma, with radiation and adjuvant (with or without concurrent) TMZ.

There is limited randomized prospective data supporting treatment efficacy in the recurrent setting, with the exception of the STELLAR trial. In this study, 343 patients with first recurrence of histologic anaplastic astrocytoma (based on the 2016 WHO classification of CNS tumors) were randomized to receive lomustine combined with eflornithine, an ornithine decarboxylase inhibitor, or lomustine alone. There was no difference in OS (the primary endpoint) between the two arms in the intention-to-treat analysis. However, in a subset analysis based on the 2021 WHO CNS5, *IDH*-mutant grade 3 (but not grade 4) astrocytomas derived clinically meaningful PFS (median 15.8 vs 7.2 months) and OS (median 34.9 vs 23.5 months) benefit in the combination arm relative to lomustine alone (Table 1) [33]. Among patients with *IDH*-mutant glioma regardless of *CDKN2A/B* status (i.e. including grade 3 and 4 tumors), OS was numerically higher in the combination arm but the difference was not statistically significant. Notable toxicities in the combination arm included hearing impairment and increased myelosuppression.

### Mutant IDH inhibitors

Oral mIDH inhibitors (mIDHi) enasidenib and ivosidenib were FDA-approved for AML in 2017 and 2018 respectively, and ivosidenib was FDA-approved for cholangiocarcinoma in 2021 [[34], [35], [36]]. Ivosidenib and vorasidenib are both orally available mIDHi studied in glioma. Ivosidenib inhibits mutant IDH1 while vorasidenib inhibits mutant IDH1 and IDH2. In phase 1 trials enrolling patients with recurrent, pre-treated grade 2–4 *IDH*-mutant glioma, both ivosidenib and vorasidenib demonstrated radiographic responses and disease stabilization, particularly in grade 2 and grade 3 non-enhancing tumors [37,38]. In a surgical window-of-opportunity study, both ivosidenib and vorasidenib decreased tumoral D-2HG concentrations by more than 90%; however, vorasidenib achieved a higher tumor-to-plasma ratio and numerically higher objective response rates (ORR), supporting its selection for evaluation in the phase 3 randomized INDIGO trial [39].

In the phase 3 INDIGO trial, 331 patients with residual or recurrent WHO grade 2 *IDH1/2*-mutant glioma were randomized to receive vorasidenib 40 mg daily or placebo [40]. Eligible patients had measurable non-enhancing disease, had received no prior therapy besides surgery, and lacked features deemed “high risk” (uncontrolled seizures, brainstem involvement, clinically relevant tumor-related functional or neurocognitive deficits). At a median follow-up of 14.2 months, the primary endpoint PFS was significantly prolonged with vorasidenib compared to placebo (median 27.7 months vs. 11.1 months, hazard ratio [HR] 0.39, 95% confidence interval [CI] 0.27–0.56, p < 0.001) [40]. Vorasidenib also significantly delayed time-to-next-intervention (TTNI). These results led to the FDA approval of vorasidenib in 2024 for patients aged 12 years and older with *IDH1/2*-mutant grade 2 glioma following surgery. Subsequent analysis of INDIGO data at median follow-up of 20.1 months confirmed the original results – PFS was significantly improved with vorasidenib compared to placebo (median not reached vs. 11.4 months, HR 0.35, 95% CI 0.25–0.49, p < 0.0001), as was TTNI [41]. Additionally, vorasidenib slowed tumor growth rate compared to placebo and may be associated with numerically lower rates of seizures. Importantly, vorasidenib was largely well tolerated. The most common grade ≥3 treatment related adverse events were reversible transaminase elevation, including alanine aminotransferase (ALT) elevation (10% with vorasidenib compared to 1% with placebo) and aspartate aminotransferase (AST) elevation (5% with vorasidenib compared to none with placebo) [41]. Other treatment-emergent adverse events (TEAEs) were predominantly grade 1–2 and occurred at similar frequencies between treatment and placebo arms. Fewer than 5% of patients discontinued treatment due to an adverse event. No differences in neurocognitive function or health-related quality of life were noted between treatment groups through the end of treatment [40,41].

Based on findings of the INDIGO trial, vorasidenib has emerged as a new first-line therapeutic option for patients with *IDH*-mutant grade 2 glioma, allowing for delay of chemoradiation and its associated long-term neurocognitive sequelae. Although INDIGO excluded patients with contrast-enhancing disease or gross-total resection, the FDA label does not restrict use based on these criteria, reflecting the broader biologic rationale for mIDH inhibition. Whether the PFS benefit observed in INDIGO will ultimately translate into an OS advantage remains unclear, and is unlikely to be definitively addressed due to the trial's crossover design.

## Emerging treatment approaches

### Investigations of vorasidenib

The results of the INDIGO study raised several important questions, including whether vorasidenib may benefit patients with higher grade tumors, prior chemoradiation, or in combination regimens. There is biological and early clinical rationale for the activity of vorasidenib as monotherapy in non-enhancing grade 3 *IDH*-mutant glioma [18,42]. In the phase 1 study of vorasidenib, several patients with grade 3 non-enhancing tumors achieved radiographic responses, with median PFS comparable to grade 2 tumors [38]. Additionally, retrospective analysis of early clinical experience with ivosidenib in *IDH*-mutant glioma also suggests no difference in disease control between grade 2 and grade 3 tumors [43]. These findings are plausible, as the histologic distinction between grade 2 and grade 3 gliomas – particularly astrocytomas — involves some subjectivity and inter-observer variation, and may have limited prognostic value [[44], [45], [46]]. Recognizing this, the 2025 National Comprehensive Cancer Network (NCCN) guidelines include vorasidenib as a treatment option (category 2B recommendation) for grade 3 newly diagnosed *IDH*-mutant astrocytoma or oligodendroglioma [47].

The role of vorasidenib alongside or following standard chemoradiation in higher grade gliomas is under investigation. Phase 3 randomized trials are under way to evaluate vorasidenib as maintenance therapy after first-line chemoradiation in *IDH*-mutant grade 2 or 3 astrocytoma (NCT06809322), and following radiation alongside monthly TMZ (and as maintenance therapy afterward) in grade 3 astrocytoma (NCT07215910) (Table 2). For *IDH*-mutant grade 4 astrocytoma, early-phase data suggest that vorasidenib monotherapy at recurrence is less beneficial, likely as these tumors harbor additional molecular alterations that render them less reliant on mIDH [38]. However, whether vorasidenib in combination with TMZ after radiation may enhance outcomes in grade 4 tumors is being evaluated in a phase 1b/2 study (NCT06478212) (Table 2).

**Table 2:** Select ongoing or unpublished clinical trials in *IDH*-mutant glioma utilizing mutant IDH inhibitors.

Intervention	Study population	Phase	NCT identifier
Vorasidenib + TMZ	Newly diagnosed *IDH*-mutant grade 3 astrocytoma, after RT and with TMZ	3	NCT07215910
Vorasidenib	Newly diagnosed *IDH*-mutant grade 2/3 astrocytoma, after RT and chemotherapy	3	NCT06809322
Vorasidenib + TMZ	❖ newly diagnosed or recurrent *IDH*-mutant grade 2–4 glioma (phase 1b) ❖ newly diagnosed *IDH1/2*-mutant grade 4 astrocytoma after RT (phase 2)	1b/2	NCT06478212
Vorasidenib + pembrolizumab	Recurrent *IDH*-mutant grade 2/3 glioma	1 (perioperative)	NCT05484622
Safusidenib	❖ *IDH1*-mutant grade 2/3 astrocytoma (high-risk features) or IDH*1*-mutant grade 4 astrocytoma (vs placebo) after chemoradiation ❖ *IDH1*-mutant grade 3 oligodendroglioma, previously untreated	3	NCT05303519
Olutasidenib + TMZ	Pediatric/Young adult newly diagnosed *IDH1*-mutant grade 3/4 glioma after RT	2	NCT06161974
LY3410738	*IDH1* R132-mutant glioma and other *IDH1/2* mutant solid tumors	1	NCT04521686
HMPL-306	*IDH1/2* mutant advanced or metastatic solid malignancy, including recurrent *IDH*-mutant glioma	1	NCT04762602

Abbreviations: *IDH* isocitrate dehydrogenase, *RT* radiation, *TMZ* temozolomide.

### Other mutant IDH inhibitors

mIDH inhibitors besides vorasidenib are in development for glioma (Table 2), and may harbor distinct efficacy and toxicity profiles. Safusidenib (DS-1001) is an orally available brain-penetrant mIDH1 R132 inhibitor that was evaluated in a phase 1 trial of 47 patients with recurrent pre-treated grade 2–4 *IDH1*-mutant glioma (16 grade 2; 22 grade 3; 7 grade 4; 2 unspecified) including 35 patients with enhancing tumors [48]. Responses were observed in 33% of non-enhancing and 17% of enhancing tumors, including two complete responses (CR) in grade 3 and grade 4 enhancing tumors. Median PFS was 10.4 weeks in enhancing tumors and not reached in non-enhancing tumors. On-treatment suppression of intratumoral D-2HG was confirmed. In a separate phase 2 study of safusidenib 250 mg twice daily in 27 patients with previously untreated WHO grade 2 *IDH1*-mutant glioma, ORR was 44.4%, median PFS was not reached, while event-free probability was 87.9% at 24 months [49]. Twenty-six patients (96%) experienced TEAEs, and 5 patients (18.5%) experienced treatment-related grade ≥3 TEAEs. Three patients had TEAEs leading to discontinuation, two of which were treatment-related transaminase elevation. TEAEs led to dose reduction in 9 (33%) and dose interruption in 16 (59.3%) patients. Most common TEAEs were grade 1–2 and most improved or resolved after dose interruption or discontinuation. The authors noted that the study was found to be non-compliant with Good Clinical Practice standards with respect to serious adverse event collection, and reported safety data are based on re-investigation of source documents according to the protocol. Altogether, safusidenib demonstrates a relatively high response rate in untreated grade 2 gliomas and measurable activity in higher grade pre-treated enhancing gliomas, although toxicities appear more frequent than reported with vorasidenib. An ongoing multi-part phase 3 trial is evaluating safusidenib in patients with *IDH1*-mutant grade 2/3 astrocytoma with high-risk features or grade 4 astrocytoma as maintenance therapy in a randomized placebo-controlled design (NCT05303519). A separate single-arm cohort within the same study is enrolling patients with residual or recurrent *IDH1*-mutant grade 3 oligodendroglioma following surgery (Table 2).

Olutasidenib (FT-2102) is another orally available brain-penetrant mIDH1 inhibitor and has received FDA approval for *IDH1*-mutant AML in 2022. In a phase 1 study of 26 patients with recurrent pre-treated mostly (88%) enhancing *IDH1*-mutant glioma grade 2–4 (4 grade 2, 15 grade 3, 7 grade 4), ORR was 8% and 2 patients with enhancing tumors achieved partial response (PR), one grade 3 and the other grade 4 [50]. On central blinded volumetric response assessment, 4 PRs and an additional 5 volumetric reductions not meeting formal PR thresholds were reported. Median PFS was 1.9 months among all evaluable patients and 16.9 months among the four patients with grade 2 glioma. Overall, olutasidenib was generally well-tolerated. Grade 3 TEAEs were reported in 11 (42%) patients, including elevated transaminase in six and hemiparesis in two patients. Treatment-related serious adverse events were reported in 3 (12%) patients including nausea/vomiting (grade 3), thrombocytopenia (grade 3), and hepatitis (grade 4). These findings suggest some activity in heavily pretreated *IDH1*-mutant glioma. A study of olutasidenib in combination with TMZ among pediatric/young-adult patients with *IDH1*-mutant high grade glioma is ongoing (NCT06161974) (Table 2).

Crelosidenib (LY3410738) is a brain-penetrant covalent mIDH1/2 inhibitor currently under clinical evaluation in solid tumors including glioma and cholangiocarcinoma. Preliminary results reported in a conference abstract among 27 patients with *IDH1/2*-mutant glioma, including 22 with enhancing tumors, demonstrated best response of 3 PRs and 9 stable disease (SD). Sustained D-2HG suppression was observed, and safety profile was favorable, with grade ≥3 TEAEs among all patients including anemia (4%), cholangitis (3%), headache (3%), decreased lymphocyte count (3%) and hyponatremia (3%).

HMPL-306 is a brain-penetrant mIDH1/2 inhibitor being evaluated in a phase 1 trial of patients with locally advanced or metastatic solid tumors. Preliminary results presented in a conference abstract from a cohort of 42 patients, including 20 with glioma (17 grade 2/3, 3 grade 4), demonstrated signals of clinical activity in lower-grade disease, with an ORR of 7.1% among 14 evaluable patients with grade 2/3 glioma and a median PFS of 20.5 months among all 17 patients [51].

### Immunotherapy

Several biologically informed therapies besides mIDH inhibitors are under investigation in clinical trials of *IDH*-mutant glioma (Table 3). Immune checkpoint blockade (ICB) is under investigation to counteract D-2HG-induced suppression of anti-tumor immunity [12], thus far with limited efficacy. In the phase 2 REVOLUMAB trial, 39 patients with recurrent *IDH*-mutant high grade glioma (grades 3–4, and grade 2 with anaplastic radiologic features) were treated with nivolumab [52]. The study failed to meet its pre-specified primary endpoint of 24-week PFS rate, which was found to be 28.2% with median PFS and OS of 1.84 months and 14.7 months respectively. As D-2HG-induced immunosuppression may be reversible with vorasidenib [39], combining immune checkpoint blockade with mIDH inhibition is a rational strategy. In a phase 2 study of 15 patients treated with recurrent *IDH1*-mutant tumors including seven with glioma, the combination of ivosidenib and nivolumab was safe however its activity was limited and comparable to ivosidenib monotherapy [53]. A phase 1/surgical window-of-opportunity study that combines vorasidenib and pembrolizumab in recurrent *IDH*-mutant glioma is ongoing (NCT05484622) (Table 3).

**Table 3:** Select ongoing or unpublished clinical trials in *IDH*-mutant glioma using agents besides mutant IDH inhibitors.

Agent	Class	Population	Phase	NCT Identifier
RT + PCV (randomized vs RT + TMZ)	Cytotoxic chemotherapy	Newly diagnosed oligodendroglioma grade 3	3	NCT00887146
IDH R132H peptide vaccine±avelumab (AMPLIFY NEOVAC)	Peptide vaccine targeting IDH R132H	Recurrent *IDH*-mutant glioma, grades 2 to 4	1	NCT03893903
PEPIDH1M vaccine (RESIST)	Peptide vaccine targeting IDH R132H	Recurrent *IDH*-mutant glioma, grade 2	1	NCT02193347
PEPIDH1M vaccine + Vorasidenib (ViCToRy)	Peptide vaccine targeting IDH R132H + mIDH1/2 inhibitor	Progressive *IDH*-mutant glioma, grade 2/3	1	NCT05609994
Niraparib	PARP 1/2 inhibitor	Progressive *IDH*-mutant, *ATRX*-mutant glioma, grades 2 to 4	0	NCT05076513
Olaparib + durvalumab	PARP 1/2 inhibitor + PD-L1 blocker	Progressive *IDH*-mutant glioma, grades 2 to 4	2	NCT03991832
Palbociclib	CDK4/6 inhibitor	Progressive *IDH*-mutant glioma, grade 3	2	NCT02530320
Abemaciclib	CDK4/6 inhibitor	Progressive primary brain tumors, including *IDH*-mutant glioma, grades 2 to 4	2	NCT03220646
Zotiraciclib	CDK9 inhibitor	Progressive *IDH*-mutant glioma, grades 2 to 4	1/2	NCT05588141
Zotiraciclib + TMZ	CDK9 inhibitor + alkylating chemotherapy	Progressive grade 3 *IDH*-mutant astrocytoma, and grade 4 glioma	1	NCT02942264
Telaglenastat + RT/TMZ	Glutaminase inhibitor	Newly diagnosed *IDH*-mutant astrocytoma grade 2-3	1b	NCT03528642
5-Azacitidine	Demethylating agent	Recurrent *IDH*-mutant glioma, grades 2/3	2	NCT03666559
5-Azacitidine + nivolumab	Demethylating agent + PD-1 blocker	Recurrent high-grade gliomas, including astrocytoma, *IDH*-mutant, grade 4	1	NCT06896110
ASTX727	Demethylating agent	Progressive nonenhancing *IDH*-mutant glioma, grades 2 to 4	1	NCT03922555

Abbreviations: *IDH* isocitrate dehydrogenase, *NCT* National Clinical Trial, *RT* radiation, *TMZ* temozolomide.

The *IDH1* R132H mutation results in a tumor-specific neoepitope that is presented by major histocompatibility complex (MHC) class II on tumor cells and induces a mutation-specific CD4^+^ T-cell response [54]. This finding has led to the development of IDH1 R132H-specific vaccines as a therapeutic strategy in *IDH*-mutant glioma. In the NOA16 phase 1 study of 33 patients with newly diagnosed grade 3–4 *IDH*-mutant astrocytoma, an IDH1 R132H-specific peptide vaccine was safe and induced immune responses in 93.3% of patients across multiple MHC alleles [55]. The phase 1 multi-arm AMPLIFY-NEOVAC trial is evaluating the safety and tolerability of IDH R132H-specific peptide vaccine alone or in combination with immune checkpoint blockade (NCT03893903). The phase 1 RESIST trial is evaluating the IDH1 R132H-specific peptide vaccine PEPIDH1M alongside TMZ in patients with recurrent grade 2 *IDH*-mutant glioma (NCT02193347). In the phase 1 ViCToRy trial, patients with recurrent *IDH1* R132H-mutant grade 2–3 gliomas will be treated with the PEPIDH1M vaccine in combination with vorasidenib (NCT05609994) (Table 3).

### Poly ADP Ribose Polymerase (PARP) inhibitors

In preclinical studies, *IDH*-mutant cells are sensitive to poly(adenosine 5′-diphosphate-ribose) polymerase (PARP) inhibitors [56,57]. PARP is an enzyme involved in repairing single-strand DNA breaks. PARP inhibition or trapping (preventing release of PARP from damaged DNA sites) leads to synthetic lethality in tumors with deficient homologous recombination (e.g. *BRCA1/2* mutant tumors) [58]. In *IDH*-mutant glioma, hypermethylation downstream of D-2HG has been hypothesized to create defects in homologous recombination, termed “BRCAness”, providing a therapeutic rationale for PARP inhibitors [56]. Subsequent work has suggested that rather than deficient homologous recombination, *IDH*-mutant tumors exhibit replicative stress that is resolved in a PARP-dependent mechanism [59].

In a single arm phase 2 study of 35 patients with recurrent *IDH*-mutant high grade glioma treated with olaparib monotherapy, median PFS was 2.5 months and the PFS-6 of 31.4% did not meet its primary endpoint, though 2 patients had objective response and treatment was well-tolerated, indicating feasibility and potential activity [60]. Another single-arm study of 15 patients with recurrent enhancing *IDH1/2*-mutant gliomas treated with olaparib monotherapy did not meet its pre-specified response rate threshold for activity, though prolonged stable disease was observed in patients with grades 2 and 3 histology [61]. Preliminary results from a single-arm study of 29 patients with high grade *IDH*-mutant glioma treated with olaparib and durvalumab suggest that the combination is well-tolerated but has limited efficacy [62]. Olaparib has limited brain penetration compared to other PARP inhibitors such as niraparib, particularly in tissue with intact blood-brain barrier (BBB), which may underlie these modest outcomes [63]. In a phase 1/2 window-of-opportunity study of pamiparib combined with metronomic TMZ in recurrent *IDH*-mutant glioma, pamiparib achieved meaningful concentrations in enhancing and non-enhancing tumors. Some patients achieved prolonged PFS (median 9.7 months in those with single prior line of chemotherapy, 5.9 months in those with multiple) [64]. However, the combination failed to meet its primary endpoint of a meaningful ORR, and exhibited substantial cumulative toxicity. Notably olaparib, niraparib, and pamiparib non-selectively inhibit PARP1 and PARP2. PARP1 inhibition mediates synthetic lethality whereas PARP2 inhibition mediates hematologic toxicity. Selective PARP1 inhibitors, such as AZD9574 (NCT05417594) and NMS-03305293 (NCT04910022) are in development.

### Cell cycle and metabolic vulnerabilities

Numerous vulnerabilities among *IDH*-mutant gliomas in metabolic and cell cycle pathways have been elucidated in preclinical studies and are under early clinical investigation. One downstream consequence of D-2HG is inhibition of α-ketoglutarate-dependent branched-chain amino acid transferases, leading to depletion of intracellular glutamate and in turn reduced synthesis of the critical antioxidant glutathione. This creates a state of redox vulnerability in *IDH*-mutant cells, which become highly dependent on glutaminase-mediated conversion of glutamine to glutamate to maintain glutathione levels and buffer oxidative stress [65,66]. In preclinical models, pharmacologic inhibition of glutaminase disrupts this compensatory pathway, resulting in glutathione depletion, accumulation of reactive oxygen species, and preferential cytotoxicity in *IDH*-mutant compared to *IDH*-wild type cells. Notably, glutaminase inhibition demonstrates synergistic antitumor activity when combined with radiation and TMZ, overwhelming cellular antioxidant defenses and resulting in enhanced tumor cell death [67,68]. These findings provide a biological rationale to target glutamine metabolism, particularly in combination with radiation, in *IDH*-mutant glioma. The selective glutaminase inhibitor telaglenastat is under investigation in combination with radiation and temozolomide in a phase 1 study enrolling patients with newly diagnosed grade 2 and 3 *IDH*-mutant astrocytoma (NCT03528642).

*IDH*-mutant glioma cells also exhibit a preferential reliance on the de novo pyrimidine synthesis pathway, which can be potently suppressed by inhibition of dihydroorotate dehydrogenase (DHODH), rendering these tumors particularly vulnerable to DHODH-targeted therapy [69]. BAY2402234, a brain–penetrant DHODH inhibitor, represents an attractive therapeutic strategy for *IDH*-mutant gliomas and is in early clinical investigation (NCT05061251).

Due to accumulation of D-2HG and depletion of NADPH, *IDH*-mutant gliomas exhibit increased reliance on mitochondrial oxidative metabolism for ATP production, creating a potential therapeutic vulnerability [70,71]. Zotiraciclib is a brain penetrant CDK9 inhibitor that disrupts mitochondrial function and increases oxidative stress in *IDH*-mutant glioma models [72]. These findings have supported clinical investigation of zotiraciclib as both monotherapy (NCT05588141) and in combination with temozolomide (NCT02942264) in patients with recurrent tumors.

Another molecular target is the homozygous deletion of *CDKN2A/B*, a common late event in the evolution of *IDH*-mutant gliomas that portends poor prognosis and justifies a grade 4 designation. It provides a rationale for targeting CDK–Rb pathway dysregulation using CDK4/6 inhibitors—agents widely employed in the treatment of advanced hormone receptor–positive breast cancer [[73], [74], [75]]. Loss of *CDKN2A* results in absence of the tumor suppressor p16^(INK4A)^, leading to unchecked CDK4/6 activity, phosphorylation of retinoblastoma protein (Rb), and promotion of cell-cycle progression [76]. In preclinical models of *IDH*-mutant glioma harboring homozygous *CDKN2A* deletion, the CDK4/6 inhibitors abemaciclib and palbociclib demonstrated antitumor activity, supporting CDK4/6 inhibition as a promising therapeutic strategy in this molecular subset [77]. Palbociclib and abemaciclib are under investigation for *IDH*-mutant glioma (NCT02530320, NCT03220646) (Table 3).

Inhibition of protein arginine methyltransferase 5 (PRMT5) is also emerging as a rational therapeutic strategy under early investigation in *IDH*-mutant gliomas with biallelic *MTAP* deletion. *MTAP* is located adjacent to *CDKN2A* on chromosome 9p21, is frequently co-deleted with *CDKN2A*, and confers an adverse prognosis in *IDH*-mutant glioma [78]. MTAP loss leads to accumulation of methylthioadenosine (MTA), which inhibits PRMT5 by competing with S-adenosylmethionine (SAM), and renders *MTAP*-deleted tumor cells hypersensitive to further PRMT5 inhibition [79]. In a phase 1 study of PRMT5 inhibitor PRT811, durable complete responses were observed among patients with *IDH*-mutant glioma but not among patients with *IDH*-wild type glioma, suggesting that *IDH*-mutant tumors may be particularly susceptible to PRMT5-directed synthetic lethal strategies [80].

### Hypomethylating agents

A hallmark of *IDH*-mutant gliomas is the D-2HG-mediated glioma CpG island methylation phenotype (gCIMP) which promotes gliomagenesis [14]. One underlying mechanism is hypermethylation at CCCTC-binding factor (CTCF) binding sites, which compromises CTCF binding and allows a constitutive enhancer to aberrantly activate the oncogene *PDGFRA* [81]. Demethylating agents can restore this insulation and suppress *PDGFRA* overexpression. In preclinical models, inhibition of DNA methyltransferases can reverse aberrant promoter hypermethylation at loci involved in glial differentiation and can suppress tumor growth [[82], [83], [84]]. In a clinical study of 12 patients with progressive *IDH*-mutant glioma across all grades, the hypomethylating agent 5-azacitidine—approved for the treatment of high-risk myelodysplastic syndrome and AML—demonstrated minimal activity. No radiographic responses were observed, median PFS was 4.7 months, and median OS was 25.2 months [85]. As 5-azacitidine has limited BBB penetration, multiple ongoing studies are evaluating intrathecal 5-azacitidine in adult and pediatric primary brain tumors, including a clinical trial assessing its combination with nivolumab in adults with recurrent astrocytoma, *IDH*-mutant, CNS WHO grade 4, as well as other high-grade gliomas (NCT06896110). ASTX727 is an oral combination of decitabine, a hypomethylating agent, and cedazuridine, a cytidine deaminase inhibitor that prevents decitabine degradation in the gastrointestinal tract. ASTX727 is currently being evaluated in adult patients with progressive, non-enhancing *IDH*-mutant glioma (NCT0392255). Preliminary results from 11 patients indicate that ASTX727 is well tolerated, with stable disease representing the best observed objective response [86].

## Conclusion and future directions

The discovery of oncogenic *IDH* mutations in glioma represents a transformative advance that has reshaped glioma classification, prognostication, and therapeutic development. Vorasidenib is the first targeted therapy available for *IDH*-mutant glioma, FDA-approved in 2024 as first-line therapy for patients with grade 2 *IDH*-mutant glioma following surgery, based on results of the phase 3 INDIGO trial. Radiation and cytotoxic chemotherapy continue to play a pivotal role as efficacious standard treatments, though targeted therapy holds promise to delay chemoradiation and its associated long-term neurocognitive sequelae. *IDH*-mutant gliomas remain largely incurable, undergoing genomic evolution over time and progressing to higher-grade tumors that become less reliant on mutant IDH signaling. Treating these higher-grade tumors effectively remains a challenge in the field.

Emerging therapeutic strategies in *IDH*-mutant glioma include mIDH inhibitors as well as other biologically informed approaches. The role of vorasidenib in subsets not represented in INDIGO is under investigation. Vorasidenib may confer benefit in grade 3 non-enhancing tumors, though randomized data in this subgroup are lacking. Its potential roles in combination with chemotherapy, in higher grade tumors, and as maintenance therapy after chemoradiation are under active clinical investigation. Additional mIDH inhibitors in clinical development may offer distinct efficacy and toxicity profiles. Beyond direct mutant IDH inhibition, therapeutic approaches under investigation include immunotherapies, PARP inhibitors, hypomethylating agents, and novel strategies targeting metabolic and cell cycle pathway vulnerabilities driven by mutant IDH biology.

As clinical and molecular features such as tumor grade, contrast enhancement and molecular alterations may influence prognosis and therapeutic response, careful patient selection is essential to optimize clinical trial design and accurately identify effective therapies. BBB penetration remains an important determinant of therapeutic efficacy, particularly in non-enhancing *IDH*-mutant glioma where intact barrier may limit drug delivery despite activity in other enhancing CNS tumors. Surgical window-of-opportunity trials represent a valuable strategy to ascertain CNS penetration and target engagement, and may accelerate drug development in this disease. Continued integration of sound mechanistic insight with thoughtful clinical investigation remains critical to advancing the field, and holds promise to improve the quality of life and longevity of our patients.

## Author contributions

Writing – VN, GY, PYW.

Review and Editing – VN, GY, PYW.

## Declaration of competing interest

The authors declare the following financial interests/personal relationships which may be considered as potential competing interests:

Vihang Nakhate reports a relationship with Servier that includes: consulting or advisory. Gilbert Youssef reports a relationship with Servier that includes: consulting or advisory. Patrick Y Wen reports a relationship with Astrazeneca that includes: consulting or advisory and funding grants. Patrick Y Wen reports a relationship with Black Diamond Therapeutics Inc that includes: consulting or advisory and funding grants. Patrick Y Wen reports a relationship with Chimerix Inc that includes: consulting or advisory and funding grants. Patrick Y Wen reports a relationship with Day One Biopharmaceuticals Inc that includes: consulting or advisory. Patrick Y Wen reports a relationship with Fore Biotherapeutics that includes: consulting or advisory. Patrick Y Wen reports a relationship with Genenta that includes: consulting or advisory. Patrick Y Wen reports a relationship with GlaxoSmithKline Inc that includes: consulting or advisory. Patrick Y Wen reports a relationship with Kintara that includes: consulting or advisory. Patrick Y Wen reports a relationship with Merck & Co Inc that includes: consulting or advisory and funding grants. Patrick Y Wen reports a relationship with Mundipharma that includes: consulting or advisory. Patrick Y Wen reports a relationship with Nerviano Medical Sciences Srl that includes: consulting or advisory and funding grants. Patrick Y Wen reports a relationship with Novartis that includes: consulting or advisory and funding grants. Patrick Y Wen reports a relationship with Novocure Inc that includes: consulting or advisory. Patrick Y Wen reports a relationship with Sapience that includes: consulting or advisory. Patrick Y Wen reports a relationship with Servier that includes: consulting or advisory and funding grants. Patrick Y Wen reports a relationship with Telix Pharmaceuticals Limited that includes: consulting or advisory. Patrick Y Wen reports a relationship with Altido Therapeutics that includes: consulting or advisory. Patrick Y Wen reports a relationship with Bristol-Myers Squibb Company that includes: funding grants. Patrick Y Wen reports a relationship with Eli Lilly and Company that includes: funding grants. Patrick Y Wen reports a relationship with Erasca, Inc. that includes: funding grants. Patrick Y Wen reports a relationship with Global Coalition for Adaptive Research that includes: funding grants. Patrick Y Wen reports a relationship with Kazia that includes: funding grants. Patrick Y Wen reports a relationship with MediciNova Inc that includes: funding grants. Patrick Y Wen reports a relationship with Philogen that includes: funding grants. Patrick Y Wen reports a relationship with Quadriga that includes: funding grants. If there are other authors, they declare that they have no known competing financial interests or personal relationships that could have appeared to influence the work reported in this paper.
